# Trans-Nasion-Complex Approach for Endoscopic Modified Lothrop Procedure: Conception, Anatomy, and Technique

**DOI:** 10.3389/fsurg.2022.871635

**Published:** 2022-04-12

**Authors:** Yu Zhao, Jianfeng Liu, Dazhang Yang, Jun Han, Jianhui Zhao, Yibei Wang

**Affiliations:** Department of Otolaryngology-Head and Neck Surgery, China-Japan Friendship Hospital, Beijing, China

**Keywords:** endoscopic modified Lothrop procedure, frontal sinus surgery, endoscopic sinus surgery, endoscopic endonasal approach, Draf III procedure, frontal sinus approach

## Abstract

**Background:**

The endoscopic modified Lothrop procedure (EMLP) is an important procedure used to address frontal and anterior skull-base lesions. Two techniques were established, namely, the inside-out approach and the outside-in approach. The former technique take the frontal recess and the first olfactory filament (FOF) as key landmarks while the latter use the FOF as posterior boundary. In some cases, however, these two landmarks are not available. Therefore, we supplement the outside-in approach and named it trans-nasion-complex approach (TNCA) for EMLP that can be performed without locating these two landmarks.

**Methods:**

Two dry human skulls were used to observe the bony nasion complex. Then, five colored silicon-injected human head specimens were dissected *via* TNCA for EMLP. Finally, the outcomes of patients who underwent TNCA were reviewed.

**Results:**

The nasion complex is an osseous complex that consists of the nasion and its adjacent structures, including the bilateral root of nasal bones, nasal process of frontal bones, anterior portion of the perpendicular plate of the ethmoid bone that connects with the inferior aspect of the nasal bones, and portions of the bilateral frontal process of the maxillary bones. Surgical landmarks for TNCA include the anterior superior portion of the nasal septum, anterior margin and axilla of the middle turbinate, frontal process of the maxilla bone, nasal process of the frontal bone and upper part of the nasal bone. These structures form a “mushroom sign” during cadaveric dissection and surgery. Twenty-one patients underwent TNCA, of whom 9 had tumors and 12 had chronic rhinosinusitis with nasal polyps (CRSwNP). None of them had major complications.

**Conclusion:**

TNCA is expected to be a safe, and direct route for EMLP. Adequate understanding of the nasion complex and “mushroom sign” will be helpful to complete TNCA.

## Introduction

The endoscopically modified Lothrop procedure (EMLP) is also called the Draf III procedure and plays an important role in modern endoscopic endonasal surgery. It used to be a common surgical method to control refractory chronic rhinosinusitis (CRS), especially nasal polyps (CRSwNP) (1, 2). Nowadays, it is often utilized as a preparation for anterior skull-base tumor surgery (3, 4).

Classical EMLP has been reported in detail (1) and proven to have reliable short- and long-term outcomes, as well as low complication rates (5, 6). The first step in classical EMLP is to create the anterior septal window. This allows either the frontal recess to be accessed through either nostril. Once the frontal recess is identified and dissected, the frontal ostia can be enlarged by drilling the nasal process of the frontal bone anteriorly, the frontal septum medially, and the frontal process of the maxillary bone laterally. The same process is then repeated on the opposite side to connect the two frontal sinus cavities to obtain the maximum drainage for both frontal sinuses (7, 8). This technique is referred to as the inside-out procedure. Identifying the frontal recess is an important step during this procedure. It will be difficult when the frontal recess is occupied by a tumor or there is no connection between the frontal sinus and the nasal cavity. Moreover, this procedure often involves the use of angled endoscopes and instruments.

When the frontal drainage pathway is confused with the tumor or scar, it is recommended that the surgeon started to drill from the anterosuperior aspect of the middle turbinate attachment, as described in previous studies (9, 10). Based on the above statement, Chin et al. (11) dissected from the outside to inside aspect of the frontal ostium by removing the bone to superficial periosteum underlying the skin anteriorly, to the first olfactory filament (FOF) posteriorly, which is called the outside-in approach for EMLP.

In the current study, we investigated the bony anatomy of the nasion and adjacent areas to find reliable landmarks. This is a complement of the outside-in approach named as the trans-nasion-complex approach (TNCA), for EMLP, which can be performed without identifying the frontal recess and the FOF.

## Materials and Methods

### Anatomic Dissection

Two dry skull specimens were utilized to observe the bony nasion complex. Five colored silicon-injected anatomic fresh specimens were used to simulate the TNCA. The common carotid arteries, vertebral arteries, and internal jugular veins were isolated, cannulated, and injected with red and blue silicone. The fresh cadaver heads were dissected using rod lens endoscopes (4 mm, 18 cm, Hopkins II, 0°, Karl Storz, Tuttlingen, Germany) attached to a high-definition camera (Karl Storz Image 1S in Clara mode) and a digital video recorder system (Karl Storz AIDA). An intraoperative navigation system (Karl Storz) was used during our dissection.

### Surgical Cases

From 2016 to 2018, the authors performed 21 cases of TNCA for EMLP in our department. Institutional review board approval was obtained for the study. All of the patients were followed up for at least 2 years. Demographic information and clinical data, including pathology, outcomes and complications, were reviewed.

## Results

### Nasion Complex

**Figure 1:**
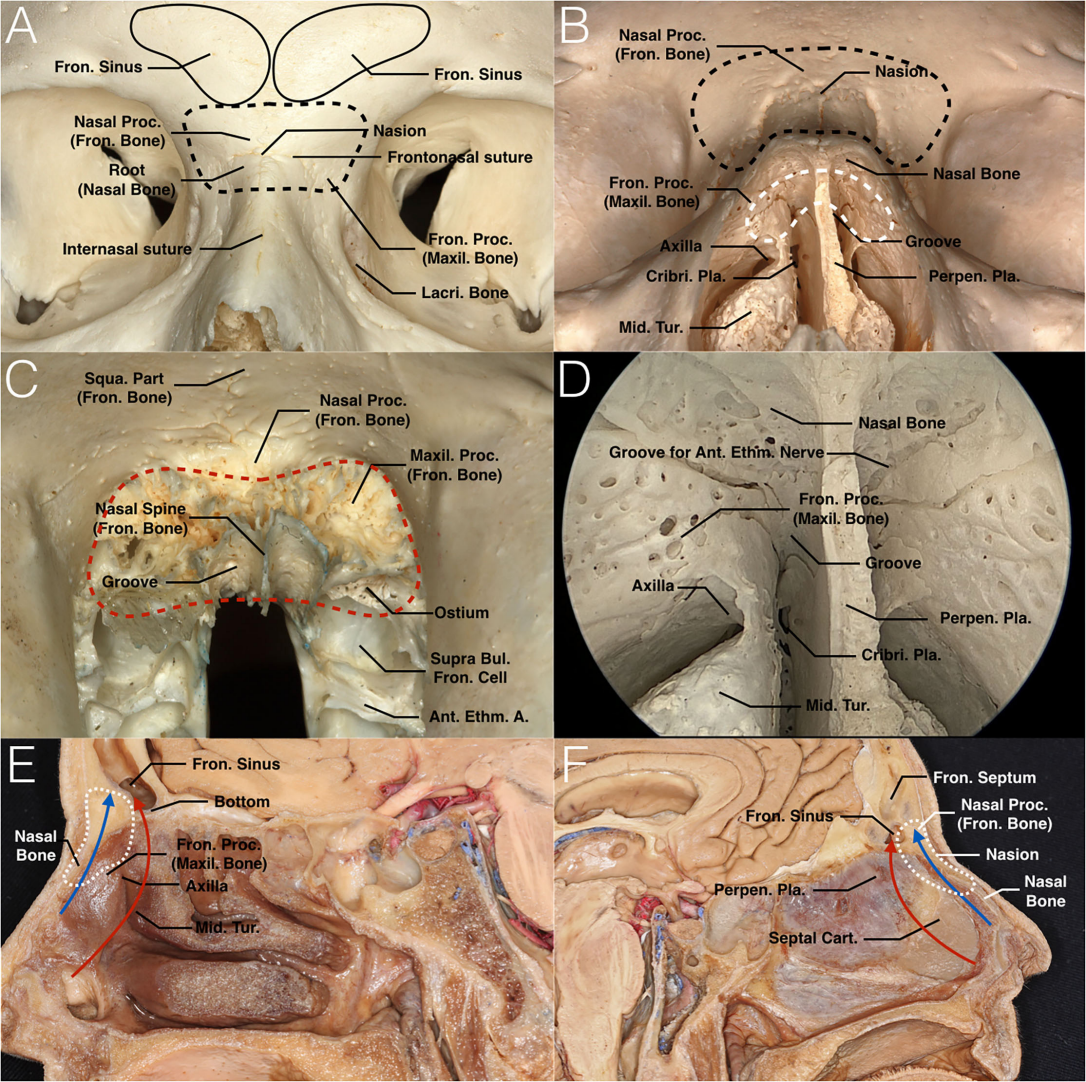
Nasion and nasion complex. **(A)** Anterior view of the surface of the nasion complex of the skull. The point at which the frontonasal and internasal sutures meet is the anthropometric landmark known as the nasion. The nasal complex consists of the nasion and its adjacent bones, including the bilateral roots of the nasal bones, nasal process of the frontal bones, perpendicular plate of the ethmoid bone that connects with the inferior aspect of the nasal bones, and part of the bilateral maxillary frontal process (black dashed curve). **(B)** Anteroinferior view of both the surface and inside portion of the nasion complex. In this image, we can see both the frontal and dorsal aspects of the nasion complex (black and white dashed curves, respectively). The latter is the main drilling area, which appears as a mushroom-shaped pattern by looking through the nasal cavity. It is bounded posteriorly by part of the perpendicular plate of the ethmoid bone that attaches to the cribriform plate. **(C)** Nasal and maxillary processes of the frontal bone are shown in anteroinferior view. The red dashed curve shows that the bone that belongs to the frontal bone and participates in the formation of the nasion complex should be removed. The frontal ostium situates posterior to the maxillary processes that connect the frontal process of maxillary bone. **(D)** Endoscopic view of the nasion complex. This image shows the junctions between these several bones on the dorsal aspect of the nasion complex. All of these junctions are located above the axilla of the middle turbinate. The groove between the nasal septum and perpendicular portion of the middle turbinate should be noted. Another groove for the anterior ethmoid nerve is on the inferior aspect of the nasal bone. **(E,F)** Sagittal views of the nasion complex and anterior skull base of the cadaveric head at the middle turbinate level **(E)** and septum level **(F)**. The traditional EMLP approach and TNCA are indicated in red and blue arrows, respectively. The former passes through the frontal recess, while the latter creates direct access to the bottom of the frontal sinus. The range of the nasion complex in the sagittal view is circled with a white dotted curve. A., artery; Ant., anterior; Bul., bulla; Cart., cartilage; Cribri., cribriform; Ethm., ethmoid; Fron., frontal; Lacri., lacrimal; Maxil., maxillary; Mid., middle; Perpen., perpendicular; Pla., plate; Proc., process; Squa., Squamous; Tur., turbinate.

The nasion is an anthropometric landmark, the point at which the frontonasal and internasal sutures meet (12, 13). The nasion complex, coined by the authors, is defined as an osseous complex that consists of the nasion and its adjacent structures, including the bilateral roots of nasal bones, nasal process of frontal bones, perpendicular plate of the ethmoid bone that connects with the inferior aspect of the nasal bones, and part of the bilateral maxillary frontal process (Figures 1A–D). It is located anteroinferiorly at the bottom of the frontal sinus and bounded superiorly by the anteroinferior wall of the frontal sinuses, inferiorly by the superior portion of the nasal bones, anteriorly by the superficial periosteum of its constituting bones, laterally by the periosteum of the orbits, and posteriorly by part of the perpendicular plate of the ethmoid bone that attaches to the cribriform plate (Figures 1E,F).

### Key Landmarks and “Mushroom Sign” of the Trans-Nasion-Complex Approach

**Figure 2:**
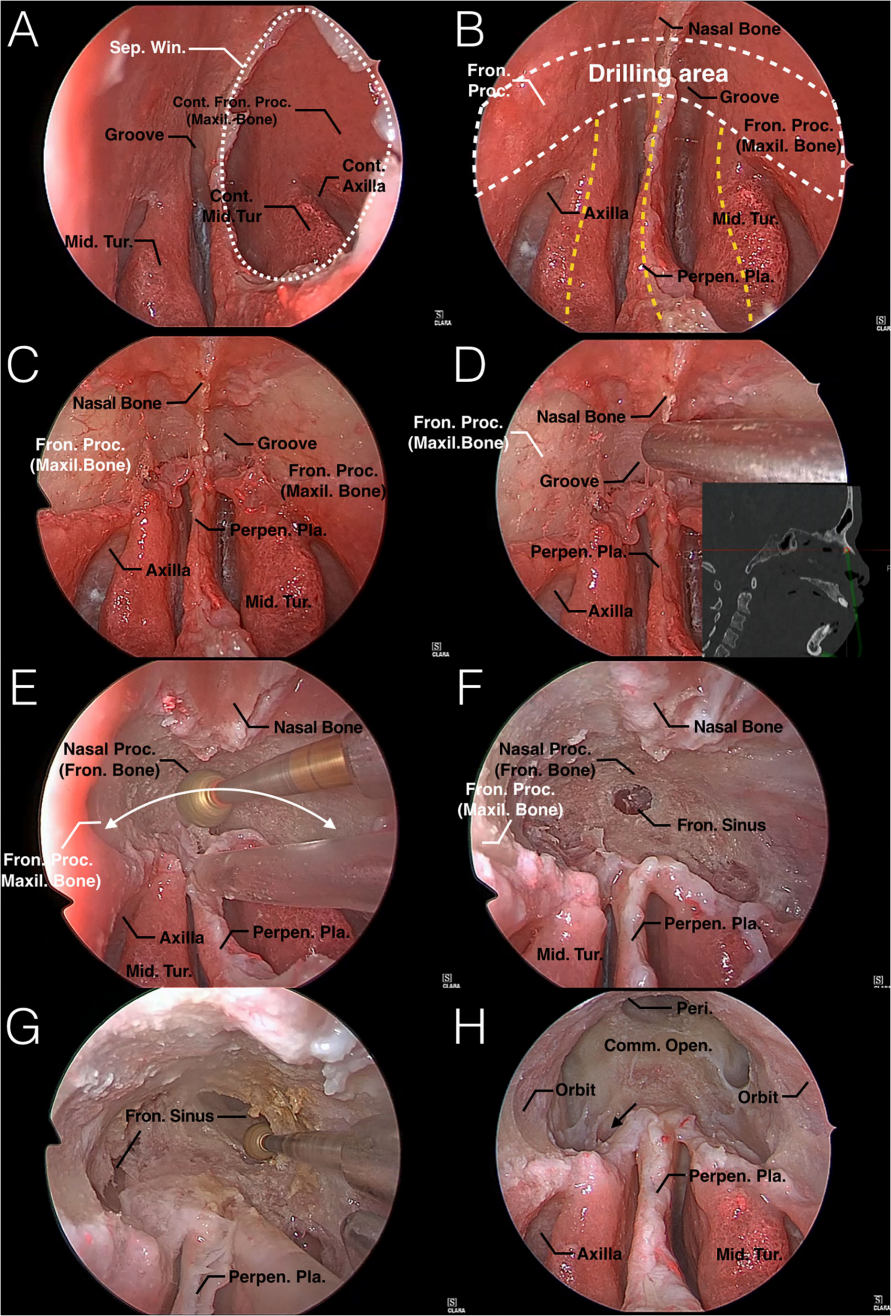
Endoscopic TNCA for EMLP: Step-by-step surgical technique in a colored silicone-injected human specimen. **(A)** A 2 × 2 cm nasal septal window is created. **(B)** The mushroom sign is fully exposed from an endoscopic perspective. The mushroom-shaped area (white dashed curve) represents the dorsal aspect of the nasion complex, which is shown in Figure 1B. The anterosuperior part of the perpendicular plate of the ethmoid bone and perpendicular portion and axilla of the bilateral middle turbinates (yellow dashed curve) resembles the stalk of a mushroom and supports the cap in the middle. According to this constant sign in the endoscopic view, surgeons can locate the nasion complex safely with these simple landmarks. **(C)** The mucosa that covers the dorsal aspect of the mushroom-shaped nasion complex is removed to show the drilling bone. Note that the naked bone area is superior and lateral to the axilla. **(D)** Access to this approach is shown by a navigation system. **(E)** Attempt to remove the nasion complex evenly in a mushroom-shaped trajectory (white curved arrow) to obtain wider access and not to drill too deep into any local area, as it may be lost and increase the incidence of complications. **(F,G)** Once the bottom mucosa of the frontal sinus has been exposed, further drilling is needed to create a common opening of the frontal sinuses. **(H)** A maximum common opening is created without exposing the FOF and frontal recesses. The mushroom pattern is still obvious after completing this approach. Notice that the supra bulla frontal cell should be opened to enlarge the anterior-posterior diameter of the common opening (black arrow). Comm., common; Cont., Contralateral; Fron., frontal; Maxil., maxillary; Mid., middle; Open., opening; Peri., Periosteum; Perpen., perpendicular; Pla., plate; Proc., process; Sep., septum; Tur., turbinate; Win., window.

The nasion complex is a critical structure that needs to be removed precisely in this approach. It presents as a mushroom-shaped pattern from an endoscopic perspective. Several reliable structures can be used as key landmarks for the nasion complex, including the anterosuperior part of the perpendicular plate of the ethmoid bone and the perpendicular portion and the axilla of the bilateral middle turbinates. They resemble the stalk of a mushroom and support the cap in the middle, which consists of upper part of the nasal bone, nasal process of the frontal bone and frontal process of the maxillary bone. This cap represents the nasion complex from an endoscopic perspective. We call this anatomic relation the “mushroom sign” (Figure 2B).

This sign is constant in endoscopic TNCA of EMLP. It was exposed in all of our cadaver dissections and operations, which helped us to locate the nasion complex safely. Moreover, nasion complex is always more anterior to the FOF according to our research, therefore making it a safer landmark to avoid cribriform plate injury.

### Trans-Nasion-Complex Approach

TNCA selects the shortest pathway that is blocked by bone, called the nasion complex. Drilling out the nasion complex consists of the nasion, and its adjacent bones create direct access to the bottom of the frontal sinus, stay away from the frontal recess and preserve the middle turbinate. Surgical access to this approach is shown in Figures 1E,F, 2D and compared with the traditional approach. A 0° endoscope and straight 5-mm diamond burr were used throughout the entire process.

#### STEP 1

Create a 2 × 2 cm window of the nasal septum. The posterior aspect of this window should be level with the anterior edge of the middle turbinate. The window can be removed superiorly to get close to the dorsal aspect of the root of the nasal bone. This maneuver should be performed as far back as possible to avoid disturbing the keystone area, which is extremely important for the aesthetic function of the nose. Then, the window should be enlarged anteriorly and inferiorly until an instrument can be passed from one side of the nasal cavity to the axilla of the middle turbinate on the opposite side. This allows either lateral boundary to be accessed through either nostril or the whole “mushroom sign” to be exposed from one endoscopic perspective (Figures 2A,B).

#### STEP 2

Remove the mucosa that covers the frontal process of the maxilla and the root of the nasal bone on both sides, which can be located with the “mushroom sign.” In particular, the mucosa that covers the groove between the nasal septum and middle turbinate should be removed. This manipulation can connect the bilateral surgical area through the nasal septal window. Laterally, the underlying osseous area is exposed 5 mm above and lateral to the axilla of the middle turbinate. Therefore, the landmark of this step is the axilla of the middle turbinate. Bone removal is always performed above and lateral to the axilla, both middle turbinates can be preserved if they are not involved (Figure 2C), or the middle turbinates should be resected in this step but still preserve the bony axilla as a key landmark. The direction to the bottom of the frontal sinus is illustrated by the intraoperative navigation system in one of our specimens (Figure 2D).

#### STEP 3

Drill the osseous area composed of the bilateral dorsal aspect of the frontal process of the maxillary bones, roots of the nasal bones, and inferior portion of the nasal process of the frontal bones from anteroinferior to superoposterior, until the bottom mucosa of frontal sinus is exposed (Figures 2E,F). Once the mucosa is in view, take this as a breakthrough point, and drill more nasal roots of frontal bones around to create a common opening between both sides of the frontal sinuses (Figure 2G). The drilling area is bounded anteriorly by the superficial periosteum of nasal bones and frontal process of maxillary bones and laterally by the periosteum of orbits.

#### STEP 4

Maximize the common opening. Remove the nasal process of frontal bone as much as possible to expand the anterior boundary. The orbital plate serves as the lateral limit of dissection. The intersinus septum is removed as high as possible. Once the above steps are completed, the posterior wall of the frontal sinus is seen with a 0° scope, which is an important landmark of the posterior boundary of this approach. The posterior wall of the frontal sinus is vertical in the upper part, and as it transforms into the anterior skull base, it will become nearly horizontal. Thus, keeping the posterior wall of the frontal sinus in view and manipulating away from the area where the posterior wall transforms into the horizontal can prevent injury to the anterior skull base. It is not necessary to deliberately expose the FOF or frontal recess in this approach. However, the supra bulla frontal cell should be opened to enlarge the anterior-posterior diameter of the common opening (Figure 2H).

### Review of Cases

**Figure 3:**
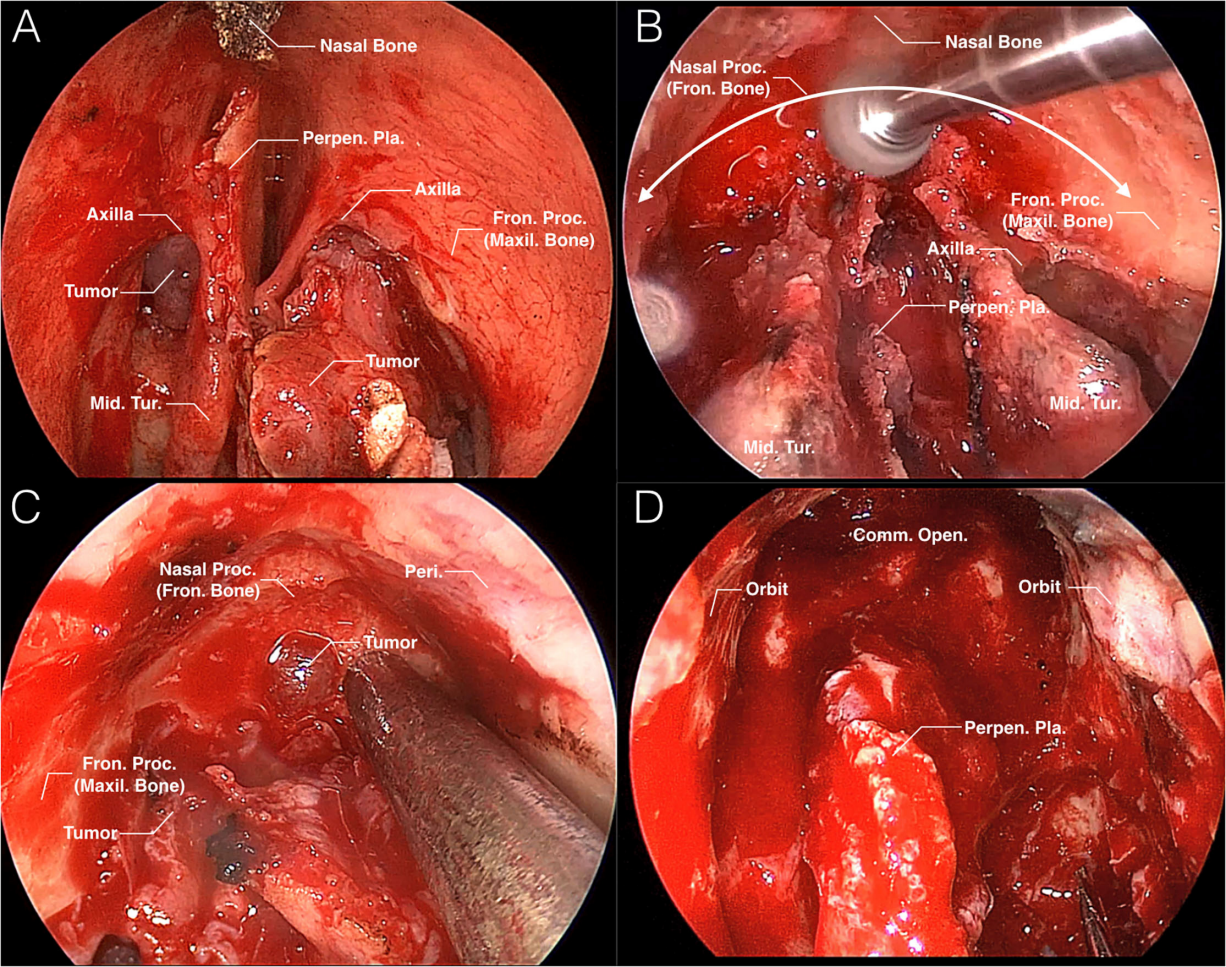
Intraoperative images of TNCA for EMLP. **(A)** Once the nasal septal window is created, the mushroom sign is fully exposed. The tumor occupies the left frontal recess and protrudes from the middle meatus. **(B)** Removal of the nasion complex is performed in a mushroom-shaped trajectory (white curved arrow). **(C)** The tumor occupying the frontal sinus is exposed. **(D)** The surgical field is shown after the TNCA of EMLP is completed. Comm., common; Fron., frontal; Maxil., maxillary; Mid., middle; Open., opening; Peri., periosteum; Perpen., perpendicular; Pla., plate; Proc., process; Tur., turbinate.

There were 21 patients with an average age of 51 years who received TNCA of EMLP. All of them were fully exposed to the mushroom sign with a 0° endoscope throughout the entire surgery. Then, the nasion complex was removed in a mushroom-shaped trajectory until the bottom mucosa of the frontal sinuses or the tumor was exposed. Next, the common opening of the bilateral frontal sinuses was enlarged, EMLP was completed or the tumor was removed. The crucial steps of this approach in a surgical case are presented in Figure 3. Demographic and clinical data for the CRSwNP and tumor groups are shown in Table 1.

**Table 1:** Demographic and clinical data of the CRSwNP and tumor groups.

	**CRSwNP group (%)**	**Tumor group (%)**
Total (*N*)	9 (42.9)	12 (57.1)
Male (*N*)	5 (55.6)	8 (66.7)
Female (*N*)	4 (44.4)	4 (33.3)
Age (mean)	50.4	52.4
Revision surgery (*N*)	9 (100)	2 (16.7)
Postoperative edema (*N*)	2 (22.2)	2 (16.7)
CSF leak (*N*)	0 (0)	0 (0)
Partial stenosis (*N*)	2 (22.2)	0 (0)
Complete stenosis (*N*)	1 (11.1)	0 (0)

None of the 21 patients suffered collapse of the nasal root or bridge. Four of them had transient soft tissue edema of the nasal root. One of the four patients also developed blepharedema on the right side. All four patients had received oral prednisone acetate 0.5 mg/kg^*^d, and their edema subsided within 5 days after surgery. None of the 21 patients suffered cerebrospinal fluid (CSF) leakage after TNCA of EMLP. There was an anterior skull-base defect in four patients after resection of a malignant tumor. All of the defects were successfully repaired with a multiple-reconstruction technique. None of them had CSF leaks or central neural system infections.

In the CRSwNP group, the symptoms of nasal obstruction, purulent rhinorrhea, and headache were improved in all patients. The olfactory function of 1 patient was not significantly improved until 2 years after the operation. Three patients still had occasional symptoms of posterior rhinorrhea, but it was not purulent secretion. It could also be improved by nasal irrigation regularly.

Partial stenosis by scar tissue around the common opening developed 2 years after surgery in only 2 patients (9.5%). Even though stenosis occurred, the neo-ostium of both frontal sinuses could drain well. Another common opening was completely stenosis by recurrent polyps. All of the restenosis cases were from the CRSwNP group.

## Discussion

### Difference Between TNCA and Inside-Out/Outside-In Approach

Traditional EMLP first finds bilateral frontal recesses during surgical access while our approach selects the shortest pathway that is blocked by bone, called the nasion complex. Drilling out of the nasion complex creates direct access to the bottom of the frontal sinus, while staying away from the frontal recess and preserving the middle turbinate (Figures 2E,F). Frontal recess with complex anatomy or involvement by lesions is suitable for this approach.

The FOF was used as the posterior boundary of the traditional approach of EMLP (14). It should also be identified as early as possible to reduce the risk of anterior skull-base injury with an outside-in approach according to previous research (11, 15, 16). However, Upadhyay et al. recommended allowing for a distance of at least 4 mm anterior to the FOF to avoid cerebrospinal fluid leakage or intracranial neurovascular damage during frontal sinusotomy. They also emphasized the position of the FOF and its correlation to the frontal sinus is variable on both sides, so the FOF may not be a reliable landmark in all patients (17). Thus, it is not necessary to intentionally expose the first fascicle in the TNCA of EMLP. Instead, we drilled evenly in a mushroom-shaped trajectory inside the nasion complex which is always separated from the anterior skull base by the frontal sinus ostium. Once the cavities of the frontal sinuses are exposed, we can enlarge the frontal ostium by looking directly at the posterior wall of the frontal sinuses, which just located in front of the anterior part of the ethmoid slit and cribroethmoidal foramen. These two landmarks were regarded as the most anterior elements of the cribriform plate and the real posterior limit of EMLP (18). Moreover, some skull-base tumors can invade and destroy the olfactory fascicle. Still, using the disappearance structure as a landmark for the posterior boundary is difficult and dangerous. In our research, we did not expose the FOF intentionally in any of the 21 cases. The incidences of partial and complete restenosis were acceptable, and there was no accidental CSF leak during our TNCA of EMLP.

In fact, the range of bone removed by these three approaches is roughly the same. The TNCA and outside-in approaches avoid dissecting the frontal recess by changing the order of bone removal. In TNCA, any surgeon can precisely drill out the nasion complex with guidance of the mushroom sign, while the outside-in approach requires thorough experience. Moreover, the outside-in approach still uses the first fascicle to locate the posterior boundary, while TNCAs do not expose it intentionally.

The main differences between TNCA, the inside-out approach (traditional EMLP) and the outside-in approach are exhibited in Table 2.

**Table 2:** Differences between the inside-out approach, outside-in approach, and TNCA.

	**Inside-out approach**	**Outside-in approach**	**TNCA**
Dissection of frontal recess	Yes	No	No
Anatomic sign	None	None	Mushroom sign
Exposure of FOF	Yes	Yes	No
Posterior boundary of surgical field	FOF	FOF	Exposed posterior wall of frontal sinus and anterior skull base
Resection of middle turbinate	Yes	No	No
Resection of supra bulla frontal cell	Yes	No	Yes
Angle of endoscope	0° and 70°	0°	0°
Angle of burr	0° and 15°	15°	0°

*TNCA, Trans-nasion-complex approach; FOF, First olfactory filament*.

### Boundary Management

Identification of surgical boundaries is crucial while removing the nasion complex. In addition to avoiding mechanical damage to the periosteum of boundaries, we should prevent thermal damage while drilling closer to boundaries. In our research, there was no damage to the orbital contents or soft tissue of the nasal root in any of the cases. Transient soft tissue edema of the nasal root occurred in four patients after surgery, and one of them developed blepharedema on the right side, which may be related to the thermal energy of a drill. Therefore, more irrigation is needed to reduce the temperature when drilling the nasion complex, especially close to the boundaries. Whether this method can reduce the incidence of swelling of surrounding tissue needs to be further studied.

The nasal bone, nasal process of the frontal bone and frontal process of the maxilla bone also play an important role in maintaining the aesthetics of the nose. The inferior margin of nasal bones, upper lateral cartilage and cartilaginous dorsal septum constitute the keystone area, and excessive removal will cause a flat nose (19). Therefore, the inferior border of the nasal bone is not injured while removing the nasion complex, which contains only the upper part of the nasal bone. In this research, there were no aesthetic changes in the external nose in any of the 21 patients.

### Strategy of Revision Surgery

Sometimes, the middle turbinate and agger nasi cell have been removed in the previous surgery, therefore taking away the axilla of the middle turbinate. For these cases, we used the mushroom sign only compose of ethmoidal perpendicular plate and maxillary frontal process to locate the nasion complex. Therefore, although the middle turbinate and the axilla are important landmarks in this approach, they are not necessary. The nasion complex is regarded as the key constant landmark during surgery, and it always point to the frontal sinus.

### Surgical Details and Pitfalls

The following details should be noted during the surgery. First, the mushroom sign should be fully exposed. An inadequate septal window, especially the residual anterior edge, will smudge our lens. Second, attention should be given to the relationship between our drilling area and anatomic landmarks. When any region is soft, we should first locate the periosteum of the nasion complex and orbit by pressing the nasal root and eyeball to avoid damaging the soft tissue of the nasal root or the orbital contents. In addition, do not drill too low in the midline to injure the cribriform plate in step 3. Moreover, attention should be given to avoid extending beyond the boundary while drilling and to maintain even drilling in a mushroom-shaped trajectory, to avoid drilling too deep into any local area, or the important structures will be injured. Finally, the relationships between the drilling area and posterior wall of the frontal sinus should always be observed in step 4. An intraoperative navigation system can help us to complete the operation more safely.

### Limitations

This paper mainly discusses the anatomic conception and surgical details of TNCA of EMLP and reviews its safety and effect. To avoid thermal damage while drilling closer to boundaries, ultrasonic bone resectors may be a good choice in the future, further research should be taken to verify the efficiency of this method. Some prospective studies are needed to confirm our opinion in the future.

## Conclusion

The trans-nasion-complex approach of the endoscopically modified Lothrop procedure is a safe and quick surgical technique that expected to be used to treat refractory frontal sinusitis or anterior skull-base tumors. The most remarkable feature of this technique is entrance of the frontal sinus without dissecting the frontal recess, which may be occupied by a tumor or scar. What we need to do in this approach is to fully expose the mushroom sign, remove the bone of the nasion complex safely, and then enlarge the frontal ostium by looking directly at the frontal sinuses without intentionally exposing the first olfactory filament. Meticulous knowledge of surgical anatomy and technical expertise in endoscopic endonasal surgery is required to apply the proposed approach safely and effectively.

## Data Availability Statement

The original contributions presented in the study are included in the article/supplementary material, further inquiries can be directed to the corresponding author/s.

## Ethics Statement

Written informed consent was obtained from the individual(s) for the publication of any potentially identifiable images or data included in this article.

## Author Contributions

YZ conceived the conception of the surgical technique. YZ and JL did the anatomic dissection research, analyzed the data, and wrote the paper. JZ and YW performed enrollment of clinical cases and collected the data. DY and JH revised the paper critically for important intellectual content. All authors read and approved the final manuscript.

## Funding

This research was supported by the Natural Science Foundation of Beijing Municipality (7212090).

## Conflict of Interest

The authors declare that the research was conducted in the absence of any commercial or financial relationships that could be construed as a potential conflict of interest.

## Publisher's Note

All claims expressed in this article are solely those of the authors and do not necessarily represent those of their affiliated organizations, or those of the publisher, the editors and the reviewers. Any product that may be evaluated in this article, or claim that may be made by its manufacturer, is not guaranteed or endorsed by the publisher.
